# 6-Shogaol, an Active Component of Ginger, Inhibits p300 Histone Acetyltransferase Activity and Attenuates the Development of Pressure-Overload-Induced Heart Failure

**DOI:** 10.3390/nu15092232

**Published:** 2023-05-08

**Authors:** Yuto Kawase, Yoichi Sunagawa, Kana Shimizu, Masafumi Funamoto, Toshihide Hamabe-Horiike, Yasufumi Katanasaka, Satoshi Shimizu, Philip Hawke, Kiyoshi Mori, Maki Komiyama, Koji Hasegawa, Tatsuya Morimoto

**Affiliations:** 1 Division of Molecular Medicine, School of Pharmaceutical Sciences, University of Shizuoka, Shizuoka 422-8526, Japan; s23807@u-shizuoka-ken.ac.jp (Y.K.); y.sunagawa@u-shizuoka-ken.ac.jp (Y.S.); kana.smz.29@gmail.com (K.S.); funamoto@u-shizuoka-ken.ac.jp (M.F.); t.hamabe@u-shizuoka-ken.ac.jp (T.H.-H.); katana@u-shizuoka-ken.ac.jp (Y.K.); mori@u-shizuoka-ken.ac.jp (K.M.);; 2 Division of Translational Research, National Hospital Organization Kyoto Medical Center, Kyoto 612-8555, Japan; nikonikomakirin@yahoo.co.jp; 3 Shizuoka General Hospital, Shizuoka 420-8527, Japan; 4 Department of Pharmacology, Institute of Biomedical Sciences, Tokushima University Graduate School, Tokushima 770-8503, Japan; 5 Laboratory of Scientific English, School of Pharmaceutical Sciences, University of Shizuoka, Shizuoka 422-8526, Japan; hawke@u-shizuoka-ken.ac.jp; 6 Graduate School of Public Health, Shizuoka Graduate University of Public Health, Shizuoka 420-0881, Japan

**Keywords:** 6-shogaol, p300, histone acetyltransferase, cardiac remodeling, heart failure

## Abstract

Hypertrophic stress-induced cardiac remodeling is a compensatory mechanism associated with cardiomyocyte hypertrophy and cardiac fibrosis. Continuation of this response eventually leads to heart failure. The histone acetyltransferase p300 plays an important role in the development of heart failure, and may be a target for heart failure therapy. The phenolic phytochemical 6-shogaol, a pungent component of raw ginger, has various bioactive effects; however, its effect on cardiovascular diseases has not been investigated. One micromolar of 6-shogaol suppressed phenylephrine (PE)-induced increases in cardiomyocyte hypertrophy in rat primary cultured cardiomyocytes. In rat primary cultured cardiac fibroblasts, 6-shogaol suppressed transforming growth factor-beta (TGF-β)-induced increases in L-proline incorporation. It also blocked PE- and TGF-β-induced increases in histone H3K9 acetylation in the same cells and in vitro. An in vitro p300-HAT assay revealed that 6-shogaol suppressed histone acetylation. The mice underwent transverse aortic constriction (TAC) surgery, and were administered 0.2 or 1 mg/kg of 6-shogaol daily for 8 weeks. 6-shogaol prevented TAC-induced systolic dysfunction and cardiac hypertrophy in a dose-dependent manner. Furthermore, it also significantly inhibited TAC-induced increases in histone H3K9 acetylation. These results suggest that 6-shogaol may ameliorate heart failure through a variety of mechanisms, including the inhibition of p300-HAT activity.

## 1. Introduction

Heart failure is the end stage of cardiovascular disease. Heart failure patients are estimated to number at least 26 million worldwide [1,2]. The prevalence of the disease is rapidly increasing in developed countries [3]. The prevalence of heart failure increases with age, with prevalence rates exceeding 10% in people older than 70 years [4,5]. A variety of factors are implicated in the development of heart failure, including increased hemodynamic pressure load caused by hypertension and other factors, regional left ventricular wall stress due to myocardial infarction, and genetic stress [6]. These chronic stresses can lead to heart failure by activating neurohumoral factors such as the sympathetic nervous system and the renin–angiotensin–aldosterone system. Heart failure drugs targeting these factors are now in clinical use [7]. Although these drug therapies have greatly improved patients’ prognoses and quality of life, the number of people suffering from heart failure and the number of deaths due to the disease continue to increase [1]. There is an urgent need for novel therapeutic drugs that inhibit the onset and progression of heart failure.

When a pressure load is applied to the heart, the heart initially adapts to it by developing concentric hypertrophy, thereby maintaining contractility [8]. Continuous pressure loading results in pressure overload, which eventually causes the heart to transition to left ventricular (LV) systolic dysfunction, beginning with left ventricular hypertrophy (LVH) [9]. The transition from LVH to LV systolic dysfunction has been reported to aggravate cardiomyocyte hypertrophy and cardiac fibrosis, including the differentiation of fibroblasts into myofibroblasts [10,11]. Therefore, inhibition of cardiomyocyte hypertrophy and cardiac fibrosis may prevent the progression from LVH to LV systolic dysfunction.

**Figure 1:**
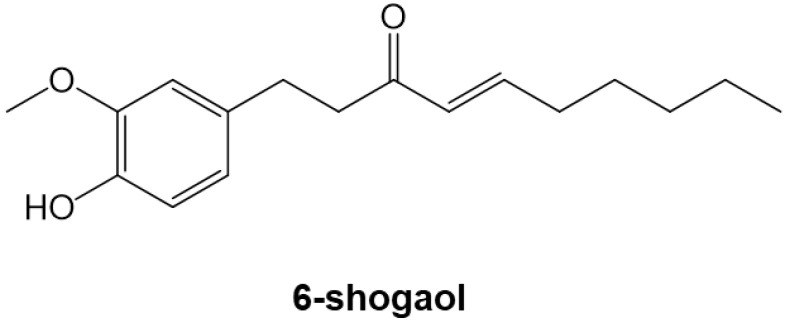
Chemical structure of 6-shogaol (1-(4-hydroxy-3-methoxyphenyl)-4-decen-3-one). The compound has an α, β-unsaturated ketone moiety.

Around the world, medicinal plants and spices are used to treat cardiovascular diseases [12,13]. An advantage of treating cardiovascular diseases with foods containing plant-derived ingredients is that they tend to be inexpensive and highly safe [14]. Ginger rhizome (Zingiber officinale Rosc) is used as a spice and as an ingredient in herbal medicines in many cultures. The major pungent component of dried ginger is the phenolic phytochemical 6-shogaol (Figure 1). It is reported to have various physiological activities, including anti-cancer, anti-inflammatory, and antioxidant effects [15]. It has recently been shown that 6-shogaol is effective against hepatocellular carcinoma, leukemia, and central nervous system diseases [16,17], but it is not yet known whether it inhibits cardiomyocyte hypertrophy, cardiac fibrosis, and the development of heart failure.

This study examined whether 6-shogaol inhibits cardiomyocyte hypertrophy and cardiac fibrosis in both primary cultured cardiomyocytes and cardiac fibroblasts. We found that the compound inhibited cardiomyocyte hypertrophy and cardiac fibrosis by inhibiting p300-HAT activity in cultured cells. Furthermore, we found that it inhibited the development of heart failure induced by pressure overload in mice. These results suggest that 6-shogaol is a potential treatment for heart failure.

## 2. Materials and Methods

### 2.1. Materials

6-shogaol was purchased from Adooq Bioscience (Irvine, CA, USA) (Figure 1). Dimethyl sulfoxide was used to dissolve the compound, and it was then stored at −20 °C. Phenylephrine (PE) was purchased from Fujifilm Wako Pure Chemical Industries (Osaka, Japan). TGF-β was purchased from Peprotech (Cranbury, NJ, USA). Each solution was filtered with a 0.45 µm Millipore filter (Merck Millipore Ltd., Cork, Ireland) and stored at −20 °C.

### 2.2. Animal Experiments

Male Sprague Dawley (SD) rats and C57BL/6j male mice were purchased from Japan SLC (Shizuoka, Japan) and CREA Japan (Tokyo, Japan), respectively. All animal experiments were carried out in accordance with the guidelines on animal experiments of the University of Shizuoka (number 156161) and Kyoto Medical Center (number 27-26-2) following protocols approved by the ethics committees of the institutions.

### 2.3. Cardiomyocyte and Cardiac Fibroblast Culture

Cardiomyocytes and cardiac fibroblasts were isolated from SD rats that were 1 or 2 days of age, as previously described [18]. To the serum-free cultured media of cardiomyocytes and cardiac fibroblasts was added 6-shogaol (0.3 or 1 μM). Hypertrophic responses were induced by treatment with 30 μM PE for 48 h. 6-shogaol was added to cardiac fibroblasts, followed by stimulation with TGF-β (10 ng/mL).

### 2.4. Immunofluorescence Staining and Measurement of Cardiomyocyte Surface Area

Immunofluorescence staining was performed as described previously [19]. In short, cardiomyocytes were fixed with 3.7% paraformaldehyde for 15 min, then blocked in 1% BSA and 0.5% NP-40/TBS-Ca for 1 h. After that, they were incubated overnight with anti-myosin heavy chain (MHC) antibodies (Leica Biosystems, Wetzlar, Germany). The cells were incubated for 1 h with Alexa Fluor 555-conjugated anti-mouse IgG (Invitrogen, Carlsbad, CA, USA) and Hoechst 33258 (Dojinjo, Kumamoto, Japan). After that, 50 cardiomyocytes were selected randomly, and cell surface area was measured using ImageJ software version 1.52a (NIH, Bethesda, MD, USA).

### 2.5. Quantitative Real-Time PCR

Quantitative real-time polymerase chain reactions (QRT-PCRs) were carried out as previously described [20]. Table 1 shows the primers that were used. 18S rRNA was used as an internal control. There are sequence homologies between rats and mice in the natriuretic factor (ANF), brain natriuretic peptide (BNP), and 18S rRNA primers.

### 2.6. Measurement of L-Proline Incorporation

DNA synthesis rate was determined by measuring [3H]-thymidine in the acid-insoluble fraction of the cells [21]. Collagen synthesis was assessed by [2,3,4,5-3H]-proline incorporation into cells, as previously described [19]. After stimulating the fibroblasts with TGF-β, to these cells was added 0.5 μCi of proline (L-[2,3,4,5-3H]-proline, Moravek Biochemicals Inc., Brea, CA, USA) per well, and the cells were then cultured at 37 °C with 5% CO_2_ for 48 h. After that, the cells were washed thrice with PBS and incubated in 200 μL of 5% trichloroacetic acid (TCA, Fujifilm Wako, Osaka, Japan) for 30 min at room temperature. After the cell residues were rinsed once with 5% TCA, the cells were solubilized in 0.5 N NaOH (Fujifilm Wako) for 30 min and then collected via neutralization with an equal amount of 0.5 N HCl (Fujifilm Wako). The collected cells were mixed with 3 mL of Clear-sol II (Nacalai Tesque, Kyoto, Japan) in wells. Finally, radioactivity was measured with a scintillation counter (LSC-7400, Aloka, Tokyo, Japan) for 3 min per sample.

### 2.7. Sample Preparation and Western Blotting

Whole cell lysate (WCL) and histone extract were prepared from cultured cardiomyocytes, cardiac fibroblasts, and mouse hearts, and Western blotting was carried out as previously described [20]. In short, after the cells were harvested, WCL was prepared on ice with cell lysis buffer (50 mM Tris-HCl pH 8.0, 150 mM NaCl, 2% Nonidet P40 (Sigma-Aldrich, St. Louis, MO, USA), 0.2 mM ethylenediaminetetraacetic acid) for 5 min. Histone fractions from primary cultured cardiomyocytes and the heart tissue of TAC mice were isolated by acid extraction [22]. SDS-PAGE was used to resolve 10 μg of each fraction. In the Western blotting assays, the primary antibodies used were mouse monoclonal anti-α-SMA antibody (Sigma-Aldrich), mouse monoclonal anti-β-actin antibody (Sigma-Aldrich), rabbit polyclonal anti-histone H3 antibody, and rabbit polyclonal anti-acetyl-histone H3 (Lys9) (Cell Signaling Technology, Danvers, MA, USA); the secondary antibodies used were anti-rabbit antibody (MBL, Aichi, Japan) and anti-mouse antibody (MBL). An Amersham Imager 680 (GE Healthcare, Chicago, IL, USA) was used to visualize the blots, and ImageJ software version 1.52a (NIH) was used for quantification.

### 2.8. In Vitro p300-Histone Acetyltransferase Assay

We carried out an in vitro HAT assay as described previously [20]. Briefly, 5 μg of core histones from calf thymus (Worthington, Columbus, OH, USA) was incubated in HAT buffer (50 mM Tris-HCl pH 8.0, 10% glycerol, 0.1 mM EDTA pH 8.0, 1 mM DTT) with a purified p300-HAT recombinant domain (amino acids 1284–1673) in the presence or absence of 6-shogaol for 30 min at room temperature. Acetyl-CoA was added to each sample to initiate the reaction. After that, each sample was incubated for 60 min before being subjected to SDS polyacrylamide gel electrophoresis (SDS-PAGE). Western blotting was then carried out with anti-acetyl-histone H3 (Lys9) and anti-histone H3 antibodies. A concentration–response curve was generated in order to calculate the 50% inhibitory concentration (IC50).

### 2.9. Transverse Aortic Constriction Surgery and Drug Treatment

Transverse aortic constriction (TAC) surgery was carried out as previously described [23]. Briefly, we anesthetized C57BL/6J male 8-week-old mice with an intraperitoneal injection consisting of medetomidine (0.3 mg/kg), midazolam (4 mg/kg), and butorphanol (5 mg/kg). After that, we incised the pleura to the second rib, then used a 27-gauge needle to ligate the aortic arch with a 6–0 nylon suture ligature. Ten mice in a sham surgery group underwent the same procedure without ligation of the aorta. Atipamezole (3 mg/kg) was administered intraperitoneally for analgesia [24]. The day after the operation, the TAC mice were assigned randomly to one of three groups: 0.2 mg/kg of 6-shogaol (10 mice), 1 mg/kg of 6-shogaol (10 mice), or vehicle (0.5% CMC-Na in saline; 10 mice). 6-shogaol was suspended in 0.5% CMC-Na in saline and administered orally once a day for 8 weeks by gastric gavage.

### 2.10. Echocardiography

Eight weeks after TAC surgery, cardiac function and the outcome of the surgery were evaluated via echocardiography using a Sonos 5500 Ultrasound System using a 10–12 MHz probe (Philips, Amsterdam, The Netherlands) as previously described [25,26]. Briefly, after anesthetizing the mice with 1.0–1.5% isoflurane, two-dimensional (M-mode) images were taken of the left ventricle. Interventricular septum thickness at end-diastole (IVSd), left ventricular internal diameter end-diastole (LVIDd), and LV posterior wall diameter (LVPWd) were determined from the image. The calculation used to determine fractional shortening (FS) was [LV internal diameter end-diastole (LVIDd) − LV internal diameter end-systole (LVIDs)]/LVIDd × 100 (%). Ejection fraction (EF) was calculated as [LV internal end-diastolic volume (LVEDV) − LV end-systolic volume (LVESV)]/LVEDV × 100 (%); LV mass was calculated as 1.055 [(IVSd + LVIDd + LVPWd)^3^ − (LVIDd)^3^]; and LV mass index (LVMI) was calculated as the ratio of LV mass to body weight.

### 2.11. Histological Analysis

Masson trichrome (MT) and hematoxylin–eosin (HE) staining were carried out as described previously [26]. In brief, the excised mouse hearts were fixed in 10% formalin and embedded in paraffin, then the paraffin-embedded tissues were stained with HE and MT. The cross-sectional myocardial cell areas and perivascular fibrosis areas were assessed quantitatively as described previously [27]. ImageJ software version 1.52a (NIH) was used to measure the surface areas of 70 HE-stained cardiomyocytes. It was also used to measure the area of perivascular fibrosis in photographs of MT-stained perivascular sections. The resulting value was divided by the total area of the photograph (12,288 pixels) to provide the relative vascularized fibrosis area.

### 2.12. Statistics

Data are expressed as the mean ± SEM. Statistical comparisons were carried out using 1-way ANOVA followed by Tukey–Kramer test. A *p* value of < 0.05 was considered significant.

## 3. Results

### 3.1. 6-Shogaol Significantly Suppressed PE-Induced Hypertrophic Responses in Cardiomyocytes

**Figure 2:**
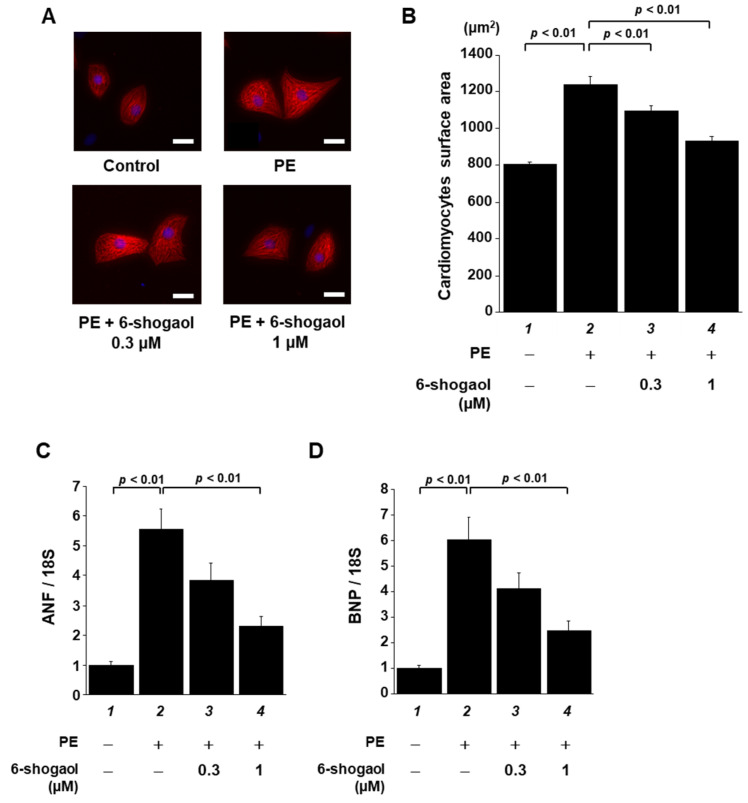
6-shogaol treatment significantly suppressed PE-induced cardiomyocyte hypertrophy in vitro. Primary cultured cardiomyocytes derived from neonatal SD rats were pretreated with 6-shogaol (0.3 or 1 μM) and then treated with the stimulant PE (30 μM) for 48 h. (**A**) Immunofluorescence staining was then carried out using anti-MHC antibody and Alexa Fluor 555-conjugated anti-mouse IgG. Scale bar: 20 μm. (**B**) ImageJ software was used to measure cell surface area (50 cells/well). The mean ± SEM values of three independent experiments are shown by the bars. The mRNA levels of hypertrophy-related ANF (**C**) and BNP (**D**) gene transcriptions were examined by quantitative RT-PCR. The mean ± SEM of four individual experiments is shown by the bars.

To investigate whether 6-shogaol suppresses cardiomyocyte hypertrophy, neonatal rat cultured cardiomyocytes were treated with 0.3 or 1 µM 6-shogaol for 2 h, then stimulated with 30 µM PE. Forty-eight hours after the treatment, cardiomyocytes were stained with anti-MHC antibody, and then cell area was measured. The results of immunofluorescence staining showed that 1 µM 6-shogaol significantly suppressed cardiomyocyte hypertrophy in a dose-dependent manner (Figure 2A,B). To investigate the effect of 6-shogaol on the transcriptional activity of hypertrophy-related genes, total RNA was isolated from these cells and then subjected to quantitative RT-PCR. The results show that 6-shogaol significantly inhibited PE-induced increases in ANF and BNP transcription, which are indicators of cardiomyocyte hypertrophy (Figure 2C,D).

### 3.2. 6-Shogaol Significantly Suppressed TGF-β-Induced Collagen Synthesis and Fibroblast Differentiation in Cardiac Fibroblasts

**Figure 3:**
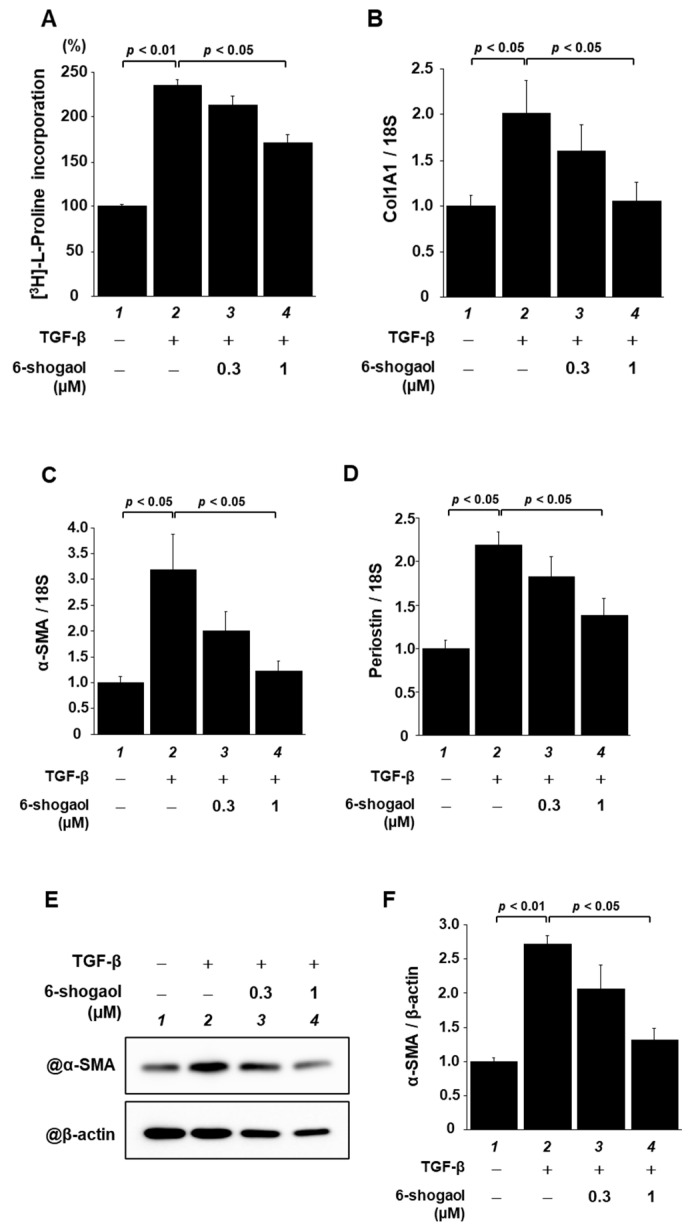
6-shogaol treatment significantly suppressed TGF-β-induced fibrotic responses in cultured cardiac fibroblasts. Primary cultured cardiac fibroblasts derived from neonatal SD rats were pretreated with 6-shogaol (0.3 or 1 μM), then stimulated with TGF-β (10 ng/mL) for 24 h or 48 h. (**A**) [3H]-L-proline incorporation that was induced by TGF-β was investigated by liquid scintillation counter. (**B**–**D**) At 24 h after stimulation, quantitative RT-PCR was used to examine the mRNA levels of fibrosis-related gene transcription of α-SMA, periostin, and Col1a1. WCL was extracted from cardiac fibroblasts after 24 h of TGF-β stimulation. (**E**) Representative image of Western blotting and (**F**) quantified α-SMA levels. All panels show the mean ± SEM of four individual experiments.

Cardiac fibroblasts, along with cardiomyocytes, are involved in cardiac function. They are considered to be dominant factors in the development of heart failure [28]. We investigated the effects of 6-shogaol on cardiac fibroblasts. Cultured cardiac fibroblasts prepared from neonatal rats were exposed to 0.3 or 1 µM 6-shogaol for 2 h, followed by stimuli with 10 ng/mL of transforming growth factor-β (TGF-β). Immediately after stimulation with TGF-β, [3H]-L-proline was added. Forty-eight hours after the treatment, incorporation of [3H]-L-proline into the cells was measured. The results show that 6-shogaol significantly reversed a TGF-β-induced increase in [3H]-L-proline incorporation (Figure 3A). To determine whether 6-shogaol affects collagen synthesis and the differentiation of cardiac fibroblasts into myofibroblasts, total RNA was isolated from these cells and subjected to quantitative RT-PCR. 6-shogaol significantly suppressed TGF-β-induced upregulation of the mRNA levels of α-SMA, periostin, and Col1a1 (Figure 3B–D). Moreover, Western blotting using the anti-α-SMA antibody showed that 6-shogaol significantly suppressed a TGF-β-induced increase inα-SMA protein expression (Figure 3E,F).

### 3.3. 6-Shogaol Blocked PE- and TGF-β-Induced Acetylation of Histone H3K9 through the Inhibition of p300-HAT Activity

**Figure 4:**
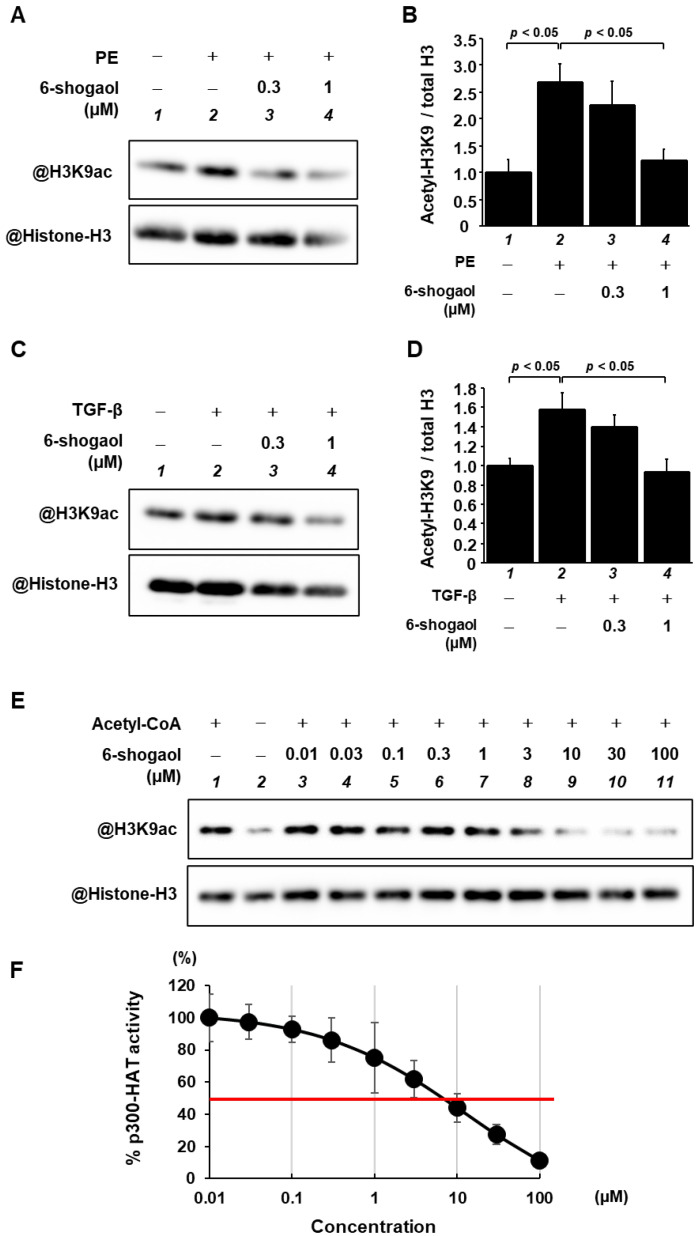
Both PE- and TGF-β-induced histone H3K9 acetylation pathways were blocked by 6-shogaol treatment. Cardiomyocytes and cardiac fibroblasts were treated with 6-shogaol (0.3 or 1 μM), followed by stimulation with 30 μM PE for 48 h or 10 ng/mL TGF-β for 6 h, respectively. (**A**,**C**) Histone fractions isolated from cardiomyocytes, and cardiac fibroblasts were subjected to Western blotting using anti-acetyl-histone H3 (Lys9) antibodies and anti-histone H3 antibodies. (**B**,**D**) Acetylated histone H3K9 and total histone H3 levels were quantified. Data are shown as the mean ± SEM of three (cardiomyocyte) and four (cardiac fibroblast) experiments. (**E**,**F**) A p300-HAT domain fragment (residues 1284–1674) was used to perform in vitro HAT assays with 6-shogaol (0.01–100 μM) or 1% DMSO as a control. (**E**) All samples were subjected to Western blotting with anti-acetyl-histone H3 (Lys9) and anti-histone H3 antibodies. (**F**) A concentration–response curve was generated by plotting acetyl-histone H3K9/histone H3 vs. log [concentrations]. The quantification is shown as the mean ± SEM of four individual experiments.

Histone acetylation is known to activate hypertrophy-related gene transcription, which then leads to the development of heart failure. To investigate the effect of 6-shogoal on histone acetylation, primary cultured cardiomyocytes were preincubated with 6-shogaol for 2 h, and subsequently either received or did not receive stimulation with 30 μM PE for 48 h. Histone fractions were isolated by acid extraction and then subjected to Western blotting to determine levels of acetylated histone H3K9 and total histone H3. The results indicate that 6-shogaol significantly suppressed PE-induced histone H3K9 acetylation in primary cultured cardiomyocytes (Figure 4A,B). Similarly, primary cultured cardiac fibroblasts were treated with saline or TGF-β in the presence or absence of 6-shogaol for 6 h. Western blotting showed that 6-shogaol almost completely blocked TGF-β-induced histone H3K9 acetylation in the fibroblasts (Figure 4C,D). Next, an in vitro HAT assay was carried out to investigate whether 6-shogaol directly suppresses p300-HAT activity (Figure 4E,F). The results reveal that p300 HAT activity is suppressed by 6-shogaol in a dose-dependent manner. The IC50 value of 6-shogaol for p300-HAT activity was 6.77 µM.

### 3.4. 6-Shogaol Significantly Attenuated Pressure-Overload-Induced Cardiac Hypertrophy and Systolic Dysfunction in Mice

**Figure 5:**
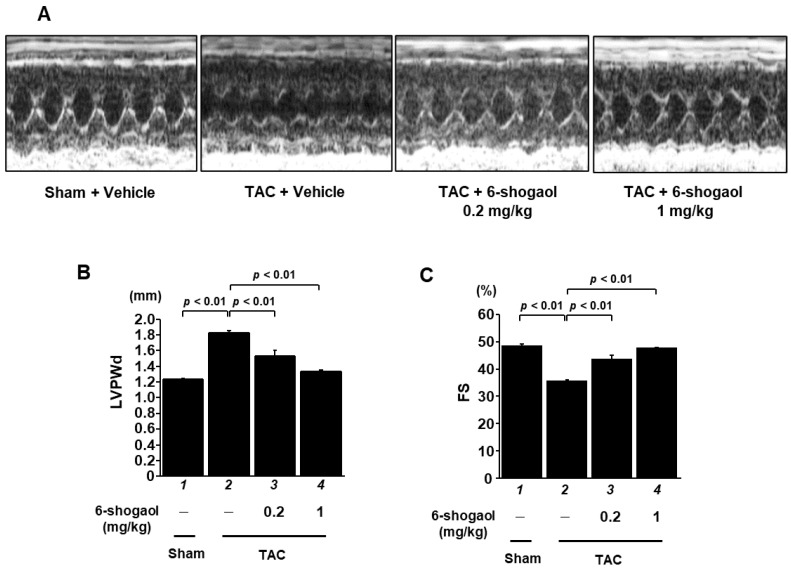
6-shogaol administration significantly reduced pressure-overload-induced systolic dysfunction in mice. Eight weeks after surgery, cardiac function was determined by echocardiography. (**A**) Representative echocardiography images (M-mode) at 8 weeks after sham or TAC surgery. The X-axis represents time; the Y-axis represents distance. (**B**,**C**) Quantified values of FS and LVPWd are shown as mean ± SEM (*n* = 10 mice).

As 6-shogaol strongly suppressed cardiomyocyte hypertrophy and cardiac fibrosis in cultured cells, it seemed probable that it would also have a preventative effect on cardiac hypertrophy and the development of heart failure in vivo. To investigate this hypothesis, C57BL/6j male mice underwent either transverse aortic coarctation (TAC) surgery, to create pressure overload, or sham surgery, as a control, followed by random assignment to daily oral administration with vehicle (10 mice), 0.2 mg/kg of 6-shogaol (10 mice), or 1 mg/kg of 6-shogaol (10 mice). Eight weeks after surgery, echocardiography was used to evaluate cardiac function (Figure 5A). The results show that LVPWd, LVSd, and LVMI, which are parameters of cardiac hypertrophy, were increased by the TAC surgery, and both 0.2 and 1 mg/kg of 6-shogaol significantly suppressed these changes (Figure 5B and Supplementary Table S1). TAC-induced decreases in FS and EF, which are parameters of cardiac function, were also significantly reduced by 6-shogaol (Figure 5C and Supplementary Table S1).

### 3.5. 6-Shogaol Significantly Inhibited Pressure-Overload-Induced Hypertrophic Responses in Mouse Heart

**Figure 6:**
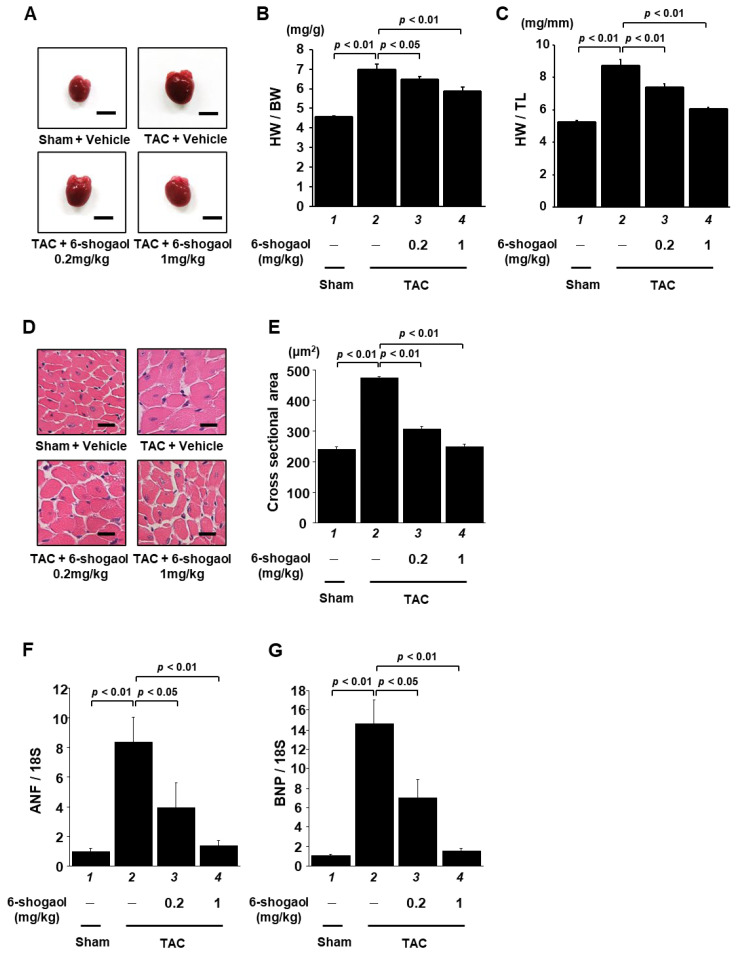
LVH was significantly inhibited by 6-shogaol administration in TAC mice. (**A**) At 8 weeks after surgery, hearts were isolated from the sham and TAC groups. Scale bar: 5 mm. Quantified HW/BW ratio (**B**) and HW/TL ratio (**C**) of mice. Results are shown as the mean ± SEM (*n* = 10 mice). (**D**) Representative photographs of HE-stained sections of LV myocardium from sham and TAC mice. Magnification: ×400. Scale bar: 10 μm. (**E**) At least 70 cells in each group were used to measure the cardiomyocyte cross-sectional area. Hypertrophy-related ANF (**F**) and BNP (**G**) gene expression levels were examined by quantitative RT-PCR. Results are shown as the mean ± SEM of six individual experiments.

After assessment via echocardiography, the mouse hearts were isolated (Figure 6A), and the effect of 6-shogaol on hypertrophic response was examined. The heart weight (Supplementary Table S1), body weight, and tibia length of each mouse were measured. Heart-weight-to-body-weight ratios (HW/BW) and heart-weight-to-tibia-length ratios (HW/TL) were calculated. The results indicate that 6-shogaol treatment reduced TAC-induced increases in HW/BW ratio (Figure 6B) and HW/TL ratio (Figure 6C) in the mice. In addition, HE staining was performed to detect pathological changes in heart tissues. The results show that the cardiomyocyte cross-sectional area was increased in mice subjected to TAC, and that both 0.2 and 1 mg/kg of 6-shogaol significantly suppressed this change (Figure 6D,E). Finally, ANF and BNP mRNA levels were assessed via quantitative RT-PCR analysis. A TAC-induced increase in mRNA levels was also inhibited by 0.2 and 1 mg/kg of 6-shogaol (Figure 6F,G).

### 3.6. 6-Shogaol Significantly Suppressed Pressure-Overload-Induced Cardiac Fibrosis in Mouse Heart

**Figure 7:**
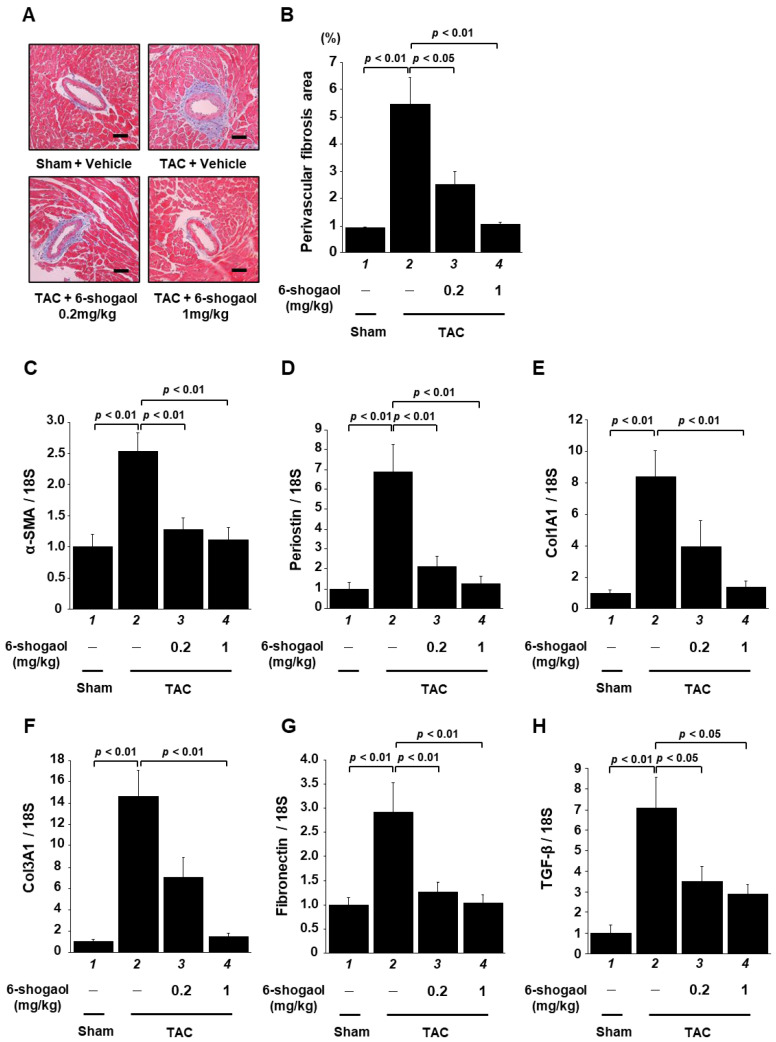
6-shogaol administration significantly suppressed cardiac fibrosis in TAC mice. (**A**) Representative images of the MT-stained perivascular fibrosis area of left ventricle myocardium in sham and TAC mice. Scale bar: 50 μm. Magnification: ×200. (**B**) For ten mice, using at least five intramyocardial coronary arteries from each mouse, the area of perivascular fibrosis in the left ventricle was measured. Results are shown as the mean ± SEM of the ten experiments. Quantitative RT-PCR was used to examine the fibrosis-related gene expression levels of α-SMA (**C**), periostin (**D**), Col1a1 (**E**), collagen3a1 (Col3a1) (**F**), fibronectin (**G**), and TGF-β (**H**). Results are shown as the mean ± SEM of ten individual experiments.

To examine the cardiac fibrosis caused by TAC surgery, MT staining was performed, followed by measurement of the area of perivascular fibrosis. The results indicate that fibrotic area was significantly increased by the TAC surgery, and that this increase was significantly suppressed by both 0.2 and 1 mg/kg of 6-shogaol in a dose-dependent manner (Figure 7A,B). Next, the mRNA levels of fibrosis-related genes were measured. Supporting the results of the histological analysis, the results show that α-SMA, periostin, Col1a1, Col3a1, fibronectin, and TGF-β levels were increased after TAC surgery, and that 6-shogaol administration significantly inhibited these increases (Figure 7C–H).

### 3.7. 6-Shogaol Significantly Suppressed a Pressure-Overload-Induced Increase in Histone Acetylation in Mouse Hearts

**Figure 8:**
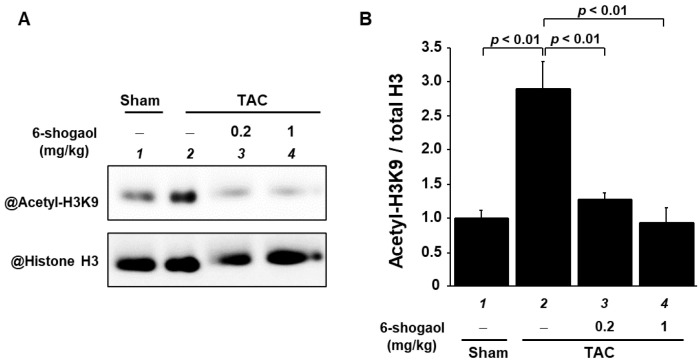
Increases in histone acetylation due to TAC surgery were significantly suppressed by 6-shogaol administration. (**A**) Western blotting was used to assess the acetylated histone H3K9 and total histone H3 levels in the histone fractions from the mouse hearts. (**B**) Quantification of levels of acetylated histone H3K9 and total histone H3. Values are shown as the mean ± SEM (*n*  =  4).

As 6-shogaol had inhibited histone H3K9 acetylation in cultured cardiomyocytes and cardiac fibroblasts, we subsequently used Western blotting to investigate whether 6-shogaol suppresses TAC-induced histone acetylation in histone fractions of the heart. The results demonstrate that the of histone H3K9 acetylation was also enhanced by TAC surgery, and that both 0.2 and 1 mg/kg of 6-shogaol significantly suppressed this increase in acetylation (Figure 8A,B).

## 4. Discussion

This study found that 6-shogaol, a ginger extract, suppressed both PE-induced cardiomyocyte hypertrophy in cardiomyocytes and TGF-β-induced differentiation into myofibroblasts in cardiac fibroblasts. Furthermore, oral administration of 6-shogaol inhibited TAC-induced LVH and progression to LV systolic dysfunction. These results demonstrate the potential of 6-shogaol as a therapy for heart failure.

An in vitro HAT assay revealed that 6-shogaol inhibited the HAT activity of p300, a common downstream signaling pathway in the nuclei of cardiomyocytes and cardiac fibroblasts. Nuclear acetylation regulated by p300-HAT activity is regarded as an important factor in pathological cardiomyocyte hypertrophy [29,30]. In addition to histones, p300 acetylates hypertrophy-responsive transcription factors, including MEF2 and GATA4, and promotes transcription of hypertrophy-related genes, including ANF, BNP, and β-MHC, thereby causing cardiomyocyte hypertrophy [31]. These findings suggest the potential of p300-HAT activity as a therapeutic target for heart failure. Our previous study has also shown that curcumin, a natural product that is structurally analogous to 6-shogaol, specifically and directly inhibits p300-HAT activity [26]. The structural similarities between 6-shogaol and curcumin include an α, β-unsaturated ketone structure in the side chain of the aromatic ring and a similar number of carbons in the side chain. On the other hand, the differences between them include the ketone structure, as well as the numbers of aromatic rings, hydrogen bond donors, and rotatable single bonds. Regarding the difference in ketone structure, while curcumin is an α, β-unsaturated diketone, 6-shogaol is an α, β-unsaturated monoketone. We have previously reported that a key factor determining the presence or absence of p300-HAT inhibitory activity is whether or not there has been a Michael addition to p300 [20]. We have also shown that the synthetic compound GO-Y030, which has an α, β-unsaturated monoketone structure, acts as a Michael acceptor in the same manner as curcumin, a diketone, and that there is no difference between these two compounds in their binding affinity to p300 [20]. In other words, it appears that 6-shogaol, like curcumin, binds directly to p300 and inhibits p300-HAT activity. While there are also some other structural differences between 6-shogaol and curcumin, the IC50 values of the two compounds for p300-HAT are almost identical, suggesting that the influence of these structural differences on p300-HAT activity is relatively minor.

In the present study, our experiment using primary cultured cardiomyocytes showed that 1 µM 6-shogaol resulted in a suppression of PE-induced cardiomyocyte hypertrophy, the transcription of hypertrophic-related genes, and the acetylation of histones. In our previous study, 10 µM curcumin blocked cardiomyocyte hypertrophy and histone acetylation in cultured cardiomyocytes, and the IC50 value of curcumin for p300-HAT activity was 9.4 µM [20]. Surprisingly, the present study found that the IC50 value of 6-shogaol for p300-HAT activity, 6.77 µM, was similar to that of curcumin, despite 6-shogaol being applied at 1/10th of the dose of curcumin. These findings suggest that 6-shogaol may inhibit cardiomyocyte hypertrophy not only through the suppression of p300-HAT activity, but also through other mechanisms. Indeed, previous work has shown that 6-gingerol, an analog of 6-shogaol lacking the α, β-unsaturated ketone structure, suppressed PE-induced cardiomyocyte hypertrophy in cultured cardiomyocytes by inhibiting the activity of the p38-MAPK pathway [32]. In other words, 6-shogaol may attenuate cardiomyocyte hypertrophy through the additive effects of the inhibition of p300-HAT activity, due both to its α, β-unsaturated ketone structure and to its signaling pathway being different from that of p300-HAT activity.

Previous studies have shown that, in the fibrotic responses of cardiac fibroblasts, TGF-β binds to the TGF-β receptor and phosphorylates Smad2/3. This phosphorylated Smad2/3 forms a complex with Smad4, and then translocates into the nucleus. In the nucleus, it binds to Smad3 and p300, resulting in increased transcription of SM22, a gene that is specific to both myofibroblasts and smooth muscle cells [33,34,35,36]. Furthermore, overexpression of HATs, such as p300 and PCAF, in embryonic fibroblast-derived 10T1/2 cells, which are a model of myofibroblast differentiation, and PAC1 cells, which are pulmonary-artery-derived smooth muscle cells, enhances histone acetylation, which then increases the promoter activity of SM22. On the other hand, the overexpression of potent HAT inhibitor molecules, such as adenovirus E1A antitumor protein and Twist1, have been reported to suppress SM22 promoter activity, as has the overexpression of HDACs [35,37]. As described above, various pathways lead to increased fibrosis, and it is thought that increased p300-HAT activity is an important factor in myofibroblast differentiation. Our experiment using primary cultured cardiac fibroblasts showed that 6-shogaol inhibited TGF-β-induced collagen deposition, fibroblast differentiation, and the acetylation of histones. A previous study of 6-gingerol, an analog of 6-shogaol that lacks the α, β-unsaturated ketone structure discussed above, showed that 6-gingerol also induced the suppression of TGF-β-induced fibrotic responses in cultured fibroblasts [32]. Comparing the results of that study with those of the present one shows that 6-shogaol has a similar inhibitory effect on fibrosis to that of 6-gingerol at approximately 1/20th the dose. This difference may be due to the inhibition of p300-HAT activity in cardiac fibroblasts caused by the α, β-unsaturated ketone of 6-shogaol. These findings indicate that 6-shogaol may suppress the differentiation of activated myofibroblasts by potently suppressing p300, which is found downstream of the TGF-β/SMAD signaling pathway, an important pathway for the enhancement of cardiac fibrosis.

The present study also found that 1 mg/kg of 6-shogaol (1) ameliorated cardiac hypertrophy and dysfunction in pressure overload model mice, (2) suppressed both the hypertrophy of individual cardiomyocytes and perivascular fibrosis, and (3) inhibited the enhancement of histone acetylation. As 6-shogaol suppressed p300-HAT activity, it may have a direct effect on p300 (Figure 4). Moreover, it has been reported to have various other activities, including antioxidation [38], ferroptosis promotion [39], and anti-inflammatory activity [15]. We hypothesize that these effects, together with the effect of p300 HAT inhibition, additively improved cardiac function.

In our previous study using transgenic (TG) mice with heart-specific overexpression of p300, we found that LV remodeling was increased to a greater extent after myocardial infarction (MI) surgery in TG mice compared to wild-type (WT) mice. However, in the TG mice, in which p300 that lacks HAT activity is specifically overexpressed in the heart, the aggravation of LV remodeling after MI surgery was comparable to that of the WT mice [29,30]. Another of our studies showed that the natural p300-specific HAT inhibitor curcumin suppressed contractile dysfunction in both MI model rats and hypertensive heart disease model rats [26]. In the present study, in comparison with curcumin, which is thought to inhibit p300-HAT activity through a similar mechanism of action to 6-shogaol [20], 6-shogaol was as effective as curcumin at 1/50th the dose. There are several possible reasons for this difference in the degree of suppression of heart failure. First, as in the case of cultured cells, 6-shogaol may have an inhibitory effect on other pathways beyond inhibiting p300-HAT activity. Second, 6-shogaol may exhibit higher bioavailability than curcumin. In a study examining the pharmacokinetics of 6-shogaol and curcumin when orally administered to rats, two indicators of bioavailability were higher for 6-shogaol than for curcumin: the area under the blood concentration–time curve (AUC) was 23 times higher, and the maximum blood concentration (C_max_) was 32 times higher [40,41]. Moreover, a previous study showed that factors affecting oral bioavailability included the number of hydrogen bond donors, the number of rotatable single bonds, and lipophilicity [42]. It is also possible that structural differences between 6-shogaol and curcumin, as with p300-HAT inhibitory activity, may be related to bioavailability.

The pharmacodynamics and pharmacokinetics of 6-shogaol are also an important consideration. When the compound is administered orally to mice, it is rapidly metabolized to a glucuronide conjugate in the liver [43], and it has been shown to have similar kinetics in clinical studies of healthy human subjects [44]. It has been reported that, after oral administration of 6-shogaol at 250 mg/kg to C57BL/6j mice, the concentration of the compound in the plasma had a T_max_ of 15 min, a C_max_ of 23.53 ng/mL, and an AUC_24h_ of 47.39 ng·h/mL. The concentration in all organs reached a maximum at 15 min after ingestion. The concentration was highest in the kidneys, followed by the intestines, liver, lungs, and heart, in that order, suggesting that 6-shogaol exerts a protective effect on these organs [45]. In the heart, tissue concentrations of 6-shogaol have been reported to remain nearly constant for up to 2 h after administration. After 2 h, most of the compound had accumulated in the stomach and kidneys, with the heart having the third highest concentration [46]. This sustained concentration in the heart may be related to the cardioprotective effects of 6-shogaol and to its suppression of the development of heart failure.

In addition, the present study revealed there to be no obvious difference in organ weight or body weight in mice treated with 6-shogaol at doses of 0.2 or 1 mg/kg for 8 weeks (Supplementary Figure S1), suggesting that the compound is unlikely to be toxic. Clinical assessment of 6-shogaol will require improvements in the low solubility and stability of 6-shogaol in an aqueous solution through formulation and the use of drug delivery systems [47].

Finally, it should be noted that there are well-known sex differences in the development and progression of cardiac hypertrophy and heart failure [48]. It has been suggested that these differences are related to hormonal [49] and chromosomal [50] factors. Regarding the TAC model used in this study, it has been reported that the heart-to-weight ratio is higher in male mice than in female mice after TAC surgery, and that genes involved in fibrosis are more highly expressed in female than in male mice [51]. Furthermore, male mice show a more severe phenotype for cardiac dysfunction [52], and many cardiovascular disease studies use male mice in their experiments [53]. The present study used exclusively male mice in order to avoid the increased complexity that would be introduced by the effect of sex differences. In the future, we intend to verify whether similar results occur in female mice.

## 5. Conclusions

This study has revealed that 6-shogaol suppresses the development of heart failure caused by LVH and LV systolic dysfunction, which are exacerbated by cardiomyocyte hypertrophy and cardiac fibrosis, by inhibiting p300-HAT activity, a downstream signaling pathway that is common to cardiomyocytes and cardiac fibroblasts, in pressure-overload-induced heart failure model mice. Clinical studies are required to assess the potential of 6-shogaol as a heart failure treatment.

## Figures and Tables

**Table 1:** Primer sequence list.

Target Gene	Forward	Reverse
ANF	ATCACCAAGGGCTTCTTCCT	CCTCATCTTCTACCGGCATC
BNP	TTCCGGATCCAGGAGAGACTT	CCTAAAACAACCTCAGCCCGT
Rat-α-SMA	TTCCGGATCCAGGAGAGACTT	TTTGGCCCATTCCAACCATC
Rat-Periostin	AGGAGCCGTGTTTGAGACCAT	CGGTGAAAGTGGTTTGCTGTTT
Rat-Col1a1	TGGTCTTGGAGGAAACTT	CCATCGTTACCACGAGCA
Mouse-α-SMA	GTCCCAGACATCAGGGAGTAA	TCGGATACTTCAGCGTCAGGA
Mouse-Periostin	ACCTGCAATGACGAAGATCC	TCACTTCTGTCACCGTTTCG
Mouse-Col1a1	AAGAAGACATCCCTGAAGTCA	TTGTGGCAGATACAGATCAAG
Mouse-Col3a1	CCCAACCCAGAGATCCCATT	GAAGCACAGGAGGTGTAGA
Mouse-Fibronectin	CCGGTGGCTGTCAGTCAGA	CCGTTCCCACTGCTGATTTATC
Mouse-TGF-β	GCTGCGCTTGCAGAGATTAAA	TTGCTGTACTGTGTGTCCAG
18S rRNA	CTTAGAGGGACAAGGGCG	GGACATCTAAGGGCATCACA

## Data Availability

The data presented in this study are available on request from the corresponding author.

## Supplementary Materials

The following supporting information can be downloaded at: https://www.mdpi.com/article/10.3390/nu15092232/s1, Table S1: Ecocardiographic parameters of sham and TAC mice. Mean ± SEM of 10 mice from each sham and TAC group. Abbreviations: HW, heart weight; IVSd, interventricular septum thickness at end-diastole; LVIDd, left ventricular internal diameter end-diastole; LVPWd, left ventricular posterior wall diameter, LVMI; left ventricular mass index; EF, ejection fraction; Figure S1: Organ weight and body weight of mice treated with 6-shogaol at doses of 0.2 or 1 mg/kg for 8 weeks. (A) Lung, (B) spleen, (C) liver, and (D) kidney were isolated from the sham and TAC groups at 8 weeks after surgery. (E) Body weight of each group was measured at 8 weeks after surgery. Data are presented as the mean ± SEM of six individual experiments.
