# Supplementary material for: 6-Shogaol, an Active Component of Ginger, Inhibits p300 Histone Acetyltransferase Activity and Attenuates the Development of Pressure-Overload-Induced Heart Failure
Source: Nutrients. 2023 May 8;15(9):2232. doi: 10.3390/nu15092232 (PMC10181444; doi:10.3390/nu15092232)
Supplement: Supplementary file 1 [file nutrients-15-02232-s001.zip › nutrients-2335168-supplementary.pdf]

nutrients

MDPI

Table S1. Ecocardiographic parameters of sham and TAC mice. Mean ± SEM of 10 mice from each sham and TAC group. Abbreviations: HW, heart weight; IVSd, interventricular septum thickness at end-diastole; LVIDd, left ventricular internal diameter end-diastole; LVPWd, left ventricular posterior wall diameter, LVMI; left ventricular mass index; EF, ejection fraction.

**Parameter**	**Sham Vehicle**	**TAC Vehicle**	**6-shogaol 0.2 mg/kg**	**6-shogaol 1 mg/kg**
**HW (mg)**	117.7 ± 1.2	185.7 ± 8.2^{**}	172.9 ± 3.8	141.1 ± $4.0^{\#\#}$
**IVSd (mm)**	1.24 ± 0.02	1.71 ± 0.05^{**}	1.45 ± $0.08^{\#}$	1.46 ± $0.04^{\#}$
**LVIDd (mm)**	2.31 ± 0.04	2.35 ± 0.1	2.57 ± 0.11	2.34 ± 0.06
**LVPWd (mm)**	1.24 ± 0.02	1.84 ± 0.02^{**}	1.56 ± $0.10^{\#\#}$	1.35 ± $0.03^{\#\#}$
**LVMI (g/mg)**	4.04 ± 0.11	7.2 ± 0.55^{**}	6.37 ± 0.82	5.23 ± $0.25^{\#}$
**EF (%)**	85.1 ± 0.79	71.2 ± 1.63^{**}	79.5 ± $1.79^{\#\#}$	84.2 ± $0.97^{\#\#}$

* $p < 0.05$, ** $p < 0.01$ vs. Sham + vehicle group. # $p < 0.05$, ## p 0.01 vs. TAC + vehicle group.

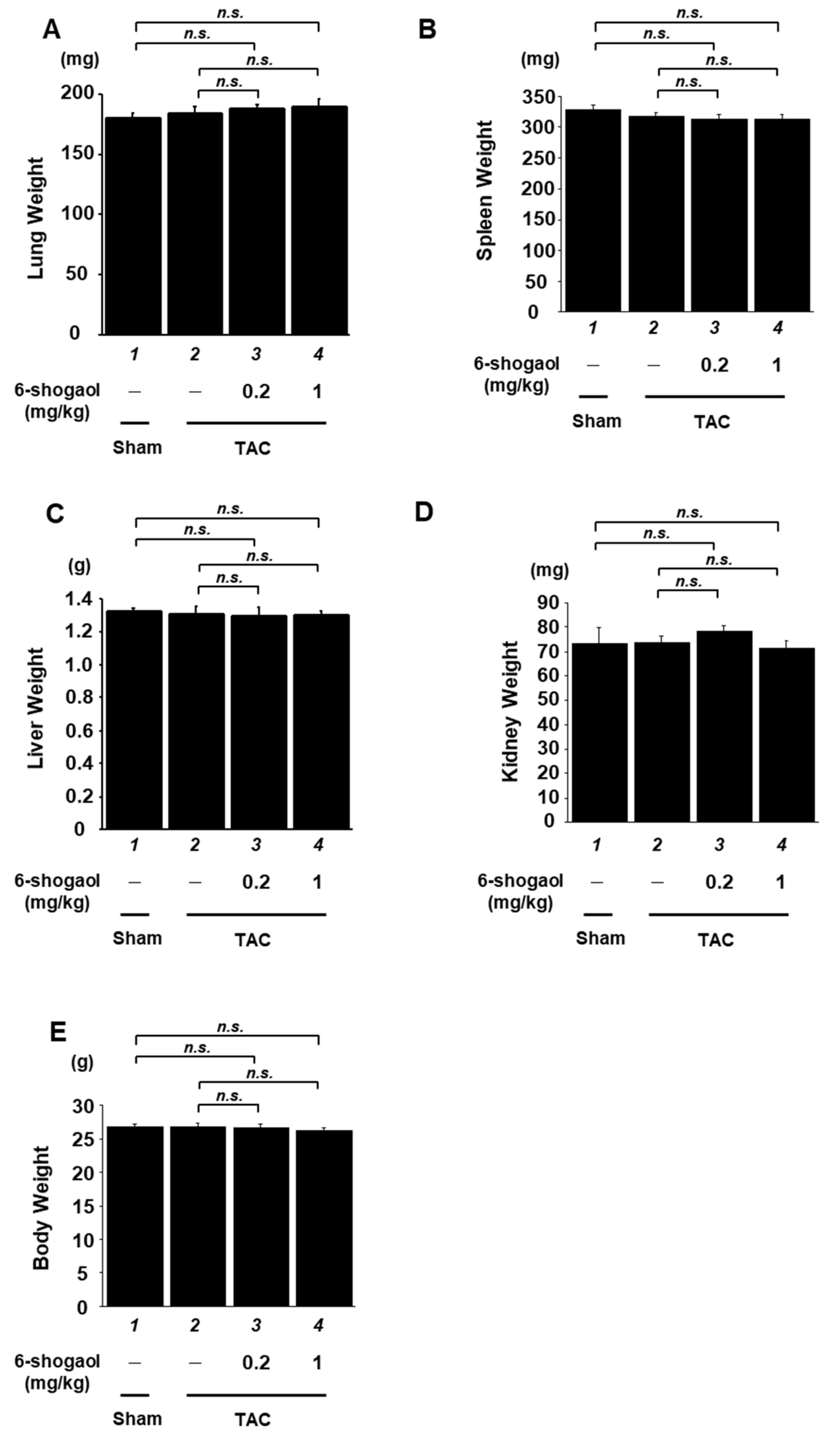

Figure S1. Organ weight and body weight of mice treated with 6-shogaol at doses of 0.2 or 1 mg/kg for 8 weeks. (**A**) Lung, (**B**) spleen, (**C**) liver, and (**D**) kidney were isolated from the sham and TAC groups at 8 weeks after surgery. (**E**) Body weight of each group was measured at 8 weeks after surgery. Data are presented as the mean ± SEM of six individual experiments.
